# *Bifidobacterium* Strains Present Distinct Effects on the Control of Alveolar Bone Loss in a Periodontitis Experimental Model

**DOI:** 10.3389/fphar.2021.713595

**Published:** 2021-09-24

**Authors:** Natali Shimabukuro, Amália C. de S. Cataruci, Karin H. Ishikawa, Bruna E. de Oliveira, Dione Kawamoto, Ellen S. Ando-Suguimoto, Emmanuel Albuquerque-Souza, Jacques R. Nicoli, Caroline M. Ferreira, Jean de Lima, Manuela R. Bueno, Leandro B. R. da Silva, Pedro H. F. Silva, Michel R. Messora, Niels O. S. Camara, Maria Regina L. Simionato, Marcia P. A. Mayer

**Affiliations:** ^1^ Department of Microbiology, Institute of Biomedical Sciences, University of São Paulo, São Paulo, Brazil; ^2^ Division of Periodontics, Department of Stomatology, School of Dentistry, University of São Paulo, São Paulo, Brazil; ^3^ Department of Microbiology, Institute of Biological Sciences, Federal University of Minas Gerais, Belo Horizonte, Brazil; ^4^ Department of Pharmaceutics Science, Institute of Environmental, Chemistry and Pharmaceutical Sciences, Federal University of São Paulo, Diadema, Brazil; ^5^ Department of Immunology, Institute of Biomedical Sciences, University of São Paulo, São Paulo, Brazil; ^6^ Department of Oral and Maxillofacial Surgery and Traumatology and Periodontology, School of Dentistry of Ribeirão Preto, University of São Paulo, Ribeirão Preto, Brazil

**Keywords:** periodontitis, *P. gingivalis*, probiotics, immune modulation, *Bifidobacterium*

## Abstract

Periodontitis is an inflammatory disease induced by a dysbiotic oral microbiome. Probiotics of the genus *Bifidobacterium* may restore the symbiotic microbiome and modulate the immune response, leading to periodontitis control. We evaluated the effect of two strains of *Bifidobacterium* able to inhibit *Porphyromonas gingivalis* interaction with host cells and biofilm formation, but with distinct immunomodulatory properties, in a mice periodontitis model. Experimental periodontitis (P+) was induced in C57Bl/6 mice by a microbial consortium of human oral organisms. *B. bifidum* 162^2A^ [B+ (1622)] and *B. breve* 110^1A^ [B+ (1101)] were orally inoculated for 45 days. Alveolar bone loss and inflammatory response in gingival tissues were determined. The microbial consortium induced alveolar bone loss in positive control (P + B-), as demonstrated by microtomography analysis, although *P. gingivalis* was undetected in oral biofilms at the end of the experimental period. TNF-α and IL-10 serum levels, and Treg and Th17 populations in gingiva of SHAM and P + B- groups did not differ. *B. bifidum* 162^2A^, but not *B. breve* 110^1A^, controlled bone destruction in P+ mice. *B. breve* 110^1A^ upregulated transcription of *Il-1β*, *Tnf-α*, *Tlr2*, *Tlr4*, and *Nlrp3* in P-B+(1101), which was attenuated by the microbial consortium [P + B+(1101)]. All treatments downregulated transcription of *Il-17*, although treatment with *B. breve* 110^1A^ did not yield such low levels of transcripts as seen for the other groups. *B. breve* 110^1A^ increased Th17 population in gingival tissues [P-B+ (1101) and P + B+ (1101)] compared to SHAM and P + B-. Administration of both bifidobacteria resulted in serum IL-10 decreased levels. Our data indicated that the beneficial effect of *Bifidobacterium* is not a common trait of this genus, since *B. breve* 110^1A^ induced an inflammatory profile in gingival tissues and did not prevent alveolar bone loss. However, the properties of *B. bifidum* 162^2A^ suggest its potential to control periodontitis.

## Introduction

Periodontitis comprises a destructive inflammatory process of the teeth supporting tissues induced by a dysbiotic subgingival microbiome (Hajishengallis, 2014). *Porphyromonas gingivalis* is considered a key stone pathogen in periodontitis, allowing the appropriate conditions to induce the disease (Kuboniwa et al., 2017), due to the ability to alter the subgingival ecosystem caused by its strategies to evade the immune system (Chopra et al., 2020). The dysbiotic microbiome of periodontitis involves not only increased levels of *P. gingivalis* (Hajishengallis, 2014), and other pathogens, but also pathobionts as *Streptococcus gordonii* (Kuboniwa and Lamont, 2010; Socransky et al., 1998), *Prevotella intermedia* (Barbosa et al., 2015)*,* and *Fusobacterium nucleatum* (Polak et al., 2017) and decreased levels of beneficial bacteria (Hajishengallis, 2014). The mechanical periodontal treatment and systemic antimicrobials are able to reduce periodontal pathogens in subgingival regions (Teughels et al., 2020). However, their effect is not entirely predictable, and long-term success requires the establishment of a program of supportive periodontal therapy following the treatment of active disease (Armitage and Xenoudi, 2016).

The ecological shift of the periodontal microbial community towards disease may be hindered by the oral administration of living beneficial bacteria with antimicrobial and immunomodulatory properties, considered as probiotics. Hence, probiotics may comprise an argued ecological therapeutic approach to control periodontitis (Matsubara et al., 2016). Probiotics may directly interfere with pathogen’s colonization by competition for adhesion sites in oral surfaces and/or in already adherent bacteria in the biofilm and production of antimicrobial substances, or indirectly, by modulating host immune response and by decreasing permeability of the epithelial barrier of mucosa surfaces (Teughels et al., 2011).

Periodontal tissues destruction is induced by an exacerbated response triggered by the dysbiotic microbiome after recognition of pathogen-associated molecular patterns (PAMPS) and damage-associated molecular pattern molecules (DAMPs) by extracellular and intracellular pattern recognition receptors (PPRs) such as Toll-Like receptors (TLRs) and nucleotide-binding oligomerization domain (NOD)-Like receptors (NLRs). Recognition by PPRs activates transcription factors, inducing the production of inflammatory cytokines and chemokines (Ojcius and Saïd-Sadier, 2012).

Periodontitis is characterized by an elevated pro-inflammatory: anti-inflammatory ratio, with increased levels of cytokines such as IL-1β, IL-6 and TNF-α (Kawamoto et al., 2020). IL-1β is a major pro-inflammatory cytokine in periodontitis (Aral et al., 2020), and its levels in gingival tissue are related to disease severity (Hou et al., 2003). IL-1β inactive precursor pro-IL-1β is only converted to its biologically active form after inflammasome activation. Inflammasomes are multiprotein complexes constituted by an intracellular receptor, an adaptor protein ASC (apoptosis-associated speck-like protein containing CARD - caspase-recruitment domain) and a pro-caspase 1 (Shibata, 2018). Receptor recognition leads to inflammasome activation, in that ASC converts pro-caspase-1 to caspase-1, which cleaves pro- IL-1β, pro-IL-18, and pro-IL-33 to their active forms, and/or induce cell death by pyroptosis (Aral et al., 2020). There are several types of inflammasome differing on their receptor molecules, activation molecules and tissue’s locations (Man et al., 2016; Abderrazak et al., 2015). NLRP3 (Nod-like receptor pyrin domain-containing protein 3) and AIM-2 (Absent in melanoma 2) inflammasomes are associated to periodontal disease, and high levels of their receptors, NRLP3 and AIM-2, are detected in gingival tissues of periodontitis patients (Aral et al., 2021).

The immune response to microbial insults in the gingival tissues also involves T cells proliferation, differentiation towards Th17 subsets, and induction of regulatory T cells (Treg) (Silva et al., 2015). Pathogens induce T cells polarization to Th17 in gingival tissues (Moutsopoulos et al., 2012), whereas inhibition of Treg increases periodontal inflammation and bone resorption (Garlet, 2010). Despite their role in homeostasis, Tregs can also differentiate into the Th17 effector subtype under inflammatory conditions, in order to mount a defense against extracellular pathogens (Round and Mazmanian, 2010).

*Bifidobacterium* are probiotics commonly used in humans (Gupta, 2011), usually considered safe (Di Gioia et al., 2014) and showed encouraging results in controlling ligature-induced periodontitis in rats by modulating the host response (Oliveira et al., 2017). In humans, the intake of *B. animalis* subsp. *lactis* led to reduced plaque and gingival indexes and decreased IL-1β levels in gingival crevicular fluid (Toiviainen et al., 2015) and was successfully used as an adjunct to the mechanical treatment of periodontitis (Invernici et al., 2018).

Certain mechanisms of probiotics are common to *Bifidobacterium* spp, such as their antimicrobial properties through the production of lactic acid (Gillor et al., 2008), which impacts *P. gingivalis* survival (Jäsberg et al., 2016). We have also shown that bifidobacteria such as *B. bifidum* 162^2A^ *and B. breve* 110^1A^ may also impair *P. gingivalis* colonization in an acid-independent mechanism, by favoring commensals over the pathogen, altering the transcription of virulence encoding genes (Ishikawa et al., 2020) and by reducing its adhesion and invasion to gingival epithelial cells (GECs) (Albuquerque-Souza et al., 2019). However, beneficial properties of probiotics are strain specific, and the most appropriate probiotic strains and their mechanisms in controlling periodontal destruction were still not determined (Mulhall et al., 2020). We previously showed that the *in vitro* overall effect of *B. bifidum* 162^2A^ on *P. gingivalis* seemed more pronounced than of *B. breve* 110^1A^, including its impact on transcription of virulence factors (Ishikawa et al., 2020). Furthermore, *B. bifidum* 162^2A^ and *B. breve* 110^1A^ also showed different effects on the production of IL-1β by *P. gingivalis* infected GECs (Albuquerque-Souza et al., 2019).

The *in vitro* determination of potential candidates is the first step to select a probiotic strain, but *in vivo* experimental data are needed to elucidate its benefits to health and underlying mechanisms. Thereby, we aimed to evaluate the effect of *B. breve* 110^1A^ and *B. bifidum* 162^2A^, with known activities over *P. gingivalis* but distinct immunomodulatory properties, in a periodontitis experimental model.

## Materials and Methods

### Animals and Group Allocation

Ninety-six, 4 weeks old C57Bl/6 male mice, bred under Specific Pathogen Free conditions were acquired from the Central Facility of School of Medicine, University of São Paulo, and maintained in the mouse breeding facility of the Department of Microbiology, Institute of Biomedical Sciences, University of São Paulo, in collective microisolators containing up to four animals, with an artificial light-dark cycle of 12 h, at a constant temperature of 22°C, and water and food available ad libidum. Animals were randomly allocated in six groups (*n* = 8), and two independent assays were performed. Experimental groups received a microbial consortium (P+) and/or *B. breve* 110^1A^ [B+ (1101)] or *B. bifidum* 162^2A^ [B+ (1622)]. Controls were inoculated with vehicles of microbial consortium (P-) and/or probiotics (B-). The groups were as follows: SHAM (P-B-) (negative control); Positive control (P + B-) (microbial consortium and probiotic vehicle); Bifidobacteria control groups P-B+ (1101) and P-B+ 1622) (microbial consortium vehicle and *B. breve* 110^1A^ or *B. bifidum* 162^2A^); and experimental groups [P + B+ (1101) and P + B+ (1622)]. The animals were monitored for weight gain, loss of mobility, and skin appearance throughout the experimental period. All procedures were performed following National Institutes of Health guidelines for experimental animal welfare and approved by the Institutional Animal Care and Use Committee (ICB USP Approval number:1112017).

### Blinding

Each animal was assigned a temporary random number within the group. Based on their position on the rack, cages were given a numerical designation. For each group, a cage was selected randomly from the pool of all cages. Blinding was carried out during the allocation, evaluation of the results, and data analysis. Blindness was unfeasible during the experiment since the same researcher prepared and inoculated the organisms. Furthermore, the bacterial suspensions differed in color from the vehicle.

### Exclusion Criteria

Animals presenting alteration in growth, weight and/or physical defects at baseline were excluded.

### Sample Size

Sample calculation was performed using alveolar bone loss as the primary outcome, based on data obtained in a pilot study. Taking into consideration a difference in the bone volume of 4,719 cubic pixels at a standard area, a sample size of 7.84 animals was adequate to obtain a Type I error rate of 5% and power greater than 80% (Charan and Kantharia, 2013). Thus, each experimental group was formed by eight animals.

### Orally Administered Cultures of Microbial Consortium and Bifidobacteria

*Bifidobacterium breve* 110^1A^ and *Bifidobacterium bifidum* 162^2A^ isolated from fecal samples of healthy children in Bahia and part of the biobank of the Federal University of Minas Gerais were tested (approval by the Ethics Committee of the Federal University of Bahia 276/2009) (Souza et al., 2013). The microbial consortium for inducing experimental periodontitis comprised: *P. gingivalis* ATCC 33277 (non-capsulated, fimbriated, genotype fimA I), *P. gingivalis* W83 (capsulated K1, afimbriated, genotype fimA IV), *Prevotella intermedia* 17 (Fukushima et al., 1992), *Fusobacterium nucleatum* ATCC 25586 (Barker et al., 1982) and *Streptococcus gordonii* DL1 (Pakula, 1965).

Bacteria from frozen stocks were cultivated in agar plates, transferred to broth, and grown to reach the stationary phase. *P. gingivalis*, *P. intermedia* and *F. nucleatum* were grown in BHI HM broth (Brain Heart Infusion Broth supplemented with 1 mg Hemin/mL and 0.1 mg Menadione/mL), in an anaerobic chamber (PlasLabs, Lansing, MI, United States) containing an atmosphere of 85% N_2_, 5%H_2_ and 10% CO_2_. *S. gordonii* was cultivated in BHI broth under microaerophilic atmosphere (10% CO_2_). *Bifidobacterium* strains were grown in MRS broth under anaerobiosis. Standard cultures were obtained for each strain, cells were harvested and resuspended in 500 µL lyophilization solution [10% skin milk with 5% L-Glutamic acid monosodium salt hydrate, and 5% dithiothreitol (Sigma-Aldrich Darmstadt, Germany)]. Aliquots were lyophilized using Freezone Triad Freezer Dryers (Freezone Triad Freezer Dryers, Labconco, Kansas City, MI, United States) at −40°C, under vacuum and maintained at −80°C. Viability was estimated for each lot under appropriate conditions.

### Experimental Treatments

Before the beginning of the experimental period, the mice resident microbiota was reduced by adding 1 mg kanamycin/mL (Gatej et al., 2018) and 1 mg amoxicillin/mL to the drinking water for 4 consecutive days, and the oral cavities were rinsed with 2% chlorhexidine digluconate (Peridex; Procter and Gamble) using a microbrush (Kang et al., 2012) for 2 days, followed by a 2-days washout period. At the first day of the experimental period, lyophilized *B. breve* 110^1A^ and *B. bifidum* 162^2A^ were suspended at 2 × 10^10^ CFU/mL in PBS/2% carboximethylcellulose gel (Gatej et al., 2018) and 50 µL aliquots were administered in the oral cavity with a gavage needle to groups [B+(1101)] or [B+(1622)]. This procedure was repeated daily for 45 days.

Lyophilized bacteria of the microbial consortium were inoculated in BHI HM broth, incubated for 6 h under anaerobiosis to recover to physiological state and suspended in PBS/2% carboximethylcellulose gel (Kang et al., 2012; Gatej et al., 2018) to reach 2 × 10^12^ CFU/mL of each strain. Viability of each strain was confirmed. 50 µL aliquots (containing 1 × 10^11^ CFU/each strain) were inoculated in the oral cavity of P+ groups 5 days/week for 5 weeks, totalizing 25 inoculations of the microbial consortium, starting at day 3 of the experimental period and ending 12 days before euthanasia. In order to avoid a direct effect of the bifidobacteria to the viable bacteria of the microbial consortium, the bifidobacteria were orally inoculated in the morning, whereas the microbial consortium was given 6 h later.

Control groups (P- and/or B-) received only vehicle (PBS/2% carboximethylcellulose gel) at the same regimen used for the infected groups (P+ and/or B+).

### Samples Collection

Forty-five days after the initial inoculation with bifidobacteria, the mice were anesthetized with ketamine/xylazine, blood was obtained by intracardiac puncture, and serum stored at −80°C. The mice were euthanized by cervical dislocation. Oral biofilm samples were obtained with sterile microbrushes and placed in Tris EDTA (TE) buffer (pH 8.0). Gingival tissue was collected from the maxilla around molars (Mizraji et al., 2013), and half transferred to RNAlater™ Stabilization Solution (Invitrogen by Thermo Fisher Scientific, Vilnius, Lithuania) for gene expression analysis and half to RPMI 1640 cell culture medium (Sigma–Aldrich, St. Louis, MO, United States) for immune cells evaluation. Then, a hemimaxilla was transferred to 4% formaldehyde solution for 24 h, transferred to PBS and stored at 4°C for alveolar bone analysis.

### Alveolar Bone Analysis

Alveolar bone resorption was determined by microtomography (MicroCT) using a microtomograph (SkyScan 1176 version 1.1, Kontich, Belgium) at 45 kV voltage, 550 uA current, 8.71 µm pixel size, 0.2 mm aluminum filter. The left hemimaxillae were scanned, and a blinded examiner selected a standard area of 60 × 30 pixel at the interproximal region between first and second M in 15 coronal sections from the second M ECJ. The images were analyzed by calculating bone volume, percentage of bone volume, and total porosity using CTAnalyser software Version 1.15.4.0, Skyscan (Rogers et al., 2007).

### Gene Expression Analysis

RNA was extracted from gingival samples using TRizol LS Reagent (Invitrogen Life Technologies, Carlsbad, CA, United States) and Mini-BeadBeater (BioSpec 3110BX Mini-BeadBeater-1 High Energy Cell Disrupter, Campinas, São Paulo, Brazil) for 20 s, twice. The resulting RNA was treated with desoxyribonuclease (Ambion™ DNase I, Invitrogen Life Technologies, Carlsbad, CA, United States). cDNA was obtained using the SuperScript^TM^ Vilo^TM^ Synthesis Kit for RT-PCR (Invitrogen Life Technologies). Quantitative PCR was performed in StepOne Plus System thermocycler (Applied Biosystems, Foster City, CA, United States). Each reaction was performed with 100 ng cDNA using TaqMan™ Gene Expression Assay (Invitrogen by Thermo Fisher Scientific, Vilnius, Lithuania). Commercial Taqman primers and probes (Invitrogen Life Technologies, Carlsbad, CA, United States) comprised Tlr-2 (Mm01213946_g1), Tlr-4 (Mm00445273_m1), Nlrp3 (Mm04210224_m1), Il-1β (Mm00434228_m1), Il-17 (Mm00439619_m1), Tnf-α Mm00607939_s1) and β-actin (Mm00607939_s1). Relative expression of target genes was calculated by the ΔΔCT method, using β-actin as endogenous control (Pfaffl, 2001), and expressed as fold changes in relation to control group (SHAM).

### *P. gingivalis* Detection in Oral Biofilm

DNA was extracted using Meta-G-Nome™ DNA Isolation kit - MGN0910 (Epicentre, Madison, WI, United States). *P. gingivalis* was detected by real-time PCR, using species-specific primer pairs (5′-TGT​AGA​TGA​CTG​ATG​GTG​AAA​ACC-3′ and 5′-ACG​TCA​CCA​CCT​CCT​TC-3′) (Amano et al., 2000). The reaction consisted of 10 ng DNA, 25 pMol of each primer, and SYBR^®^ Green Real-Time PCR Master Mix (Invitrogen Life Technologies, Carlsbad, CA, United States) and was performed at 50°C/2 min, 95°C/10 min, followed by 40 cycles at 95°C/15 s, 60°C/1 min, in a StepOne Plus thermocycler (Applied Biosystems, Foster City, CA, United States). The standard curve consisted of serially diluted *16SrRNA* of *P. gingivalis* ATCC 33277 amplicon. Efficiency was estimated as 100 ± 10%, and the data were reported as number of *P. gingivalis* 16SrRNA copies/μg DNA.

### Tregs and Th17 Cells Populations in Gingival Tissue

The percentages of CD45^+^CD3^+^CD4^+^ T cells, Foxp3^+^(Treg) or RORγt^+^ (Th17) subpopulations in gingival tissue samples were determined by flow cytometer analysis (Souto et al., 2014). Gingival samples were pooled for every four animals due to the low amount of total cells populations, and dissociated with 0.28 Wunsch/mL liverase blendyme (Gibco by Life Technologies, New York, NY, United States), using Tumor Dissociation mouse kit (MiltenyiBiotec Inc., Auburn, Al, United States) with the aid of the MACS^TM^ Octo Dissociator with Heaters (MiltenyiBiotec Inc., Auburn, Al, United States). Death cells and cell debris were distinguished from viable cells by staining with Fixable Viability Stain 570 [BD Horizon™ Fixable Viability Stain 570 (Becton; Dickinson and Company, San Diego, CA, United States)]. Then, 1–10 × 10^6^ viable cells were stained using fluorescence-bound Antibodies [APC-Cy™ seven Rat Anti-Mouse CD3, FITIC Rat Anti-Mouse CD4 and BV510 Rat Anti-Mouse CD45 (Becton; Dickinson and Company, San Diego, CA, United States)]. Cells were then permeabilized and fixed [BD Pharmingen™ Mouse Foxp3 Buffer Set kit (Becton; Dickinson and Company, San Diego, CA, United States)], and intracellular staining was performed with Alexa Fluor® 647 Rat anti-MOUSE Foxp3, e BV421 Mouse Anti-Mouse RORγt, overnight, at 4°C. Non-specific binding was blocked by BSA. Unstained samples were used as negative controls, and BD™ CompBeads (Becton; Dickinson and Company, San Diego, CA, United States) labeled used for staining compensation. Data from 100,000 events were acquired using the BD FACSCanto™ II cytometer (Becton; Dickinson and Company, San Diego, CA, United States), and analyzed using FlowJo 10.6 software (Becton; Dickinson and Company, San Diego, CA, United States).

### Serum Cytokines Levels

IL-10 and TNF-α levels in serum were evaluated by ELISA, using the BD Opteia Mouse ELISA Set kit (Becton; Dickinson and Company, San Diego, CA, United States). OD was determined at 450 nm in a spectrophotometer [Microplate Manager^®^ Software Version 5.2.1 (Bio-Rad Laboratories, INC., Hercules, CA, United States)]. After comparison to a standard curve, data were expressed in pg/mL.

### Statistical Analysis

Data were tested for normality using Kolmogorov-Smirnov test with Lilliefors correlation and homogeneity of variances was assessed by the F test. One-way ANOVA *post hoc* Tukey test was used for determining differences among the studied groups in alveolar bone parameters, relative transcription levels, percentage of Treg and Th17 cells in gingival tissues, and serum cytokines levels. Statistical significance was set at *p* < 0.05. The analyses were performed using the GraphPad Prism^®^ Version 6.0 statistical package (GraphPad software, La Jolla, CA, United States).

## Results

**FIGURE 1:**
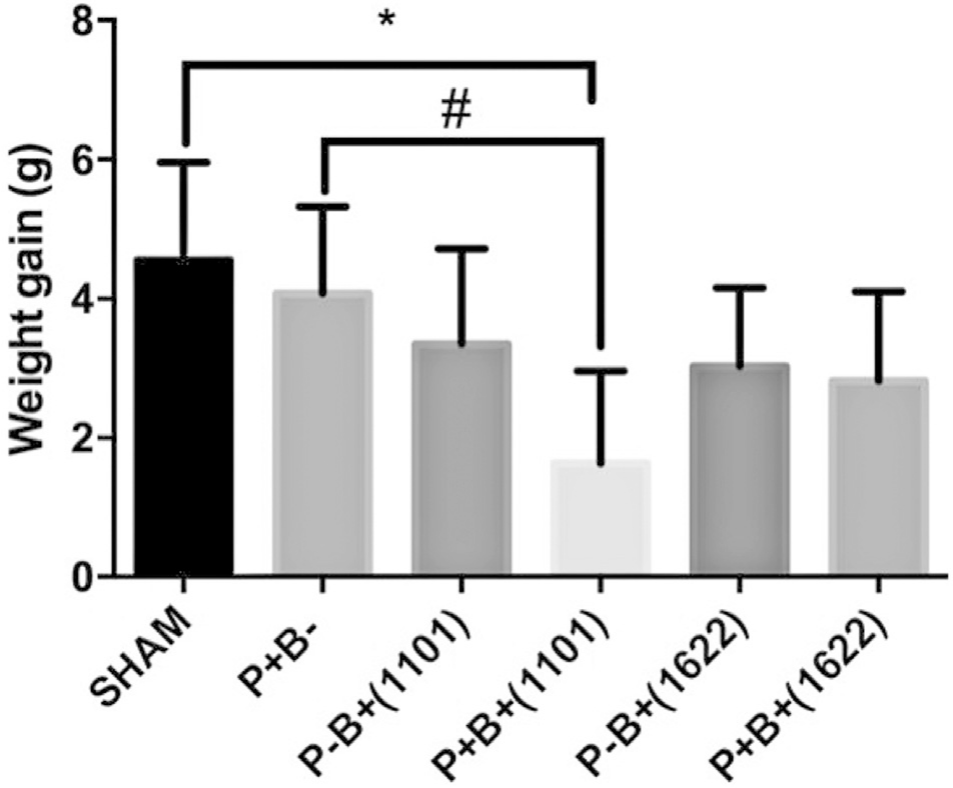
Mean and SD of weight gain in grams after 45 days of experimental period of C57Bl/6 mice submitted to different treatments: SHAM (negative control), P + B- (positive control), P-B+ (1101) (*B. breve* 110^1A^), P + B+ (1101) (microbial consortium + *B. breve* 110^1A^), P-B+ (1622) (*B. bifidum* 162^2A^) and P + B+ (1622) (Microbial consortium + *B. bifidum* 162^2A^). *Statistically significant difference in relation to negative control (SHAM), # Statistically significant difference in relation to positive control (P + B-). ANOVA, Tukey’s multiple comparison, *p* <0.05%. Data representative of two independent experiments (*n* = 8 mice/per group).

The treatments did not result in any observable alteration in skin, hair, or locomotion activity. This *in vivo* study was performed in two independent assays, which gave similar results. Weight gain was similar for all groups, except for the group which received the microbial consortium and *B. breve* 110^1A^ [P + B+ (1101)], which gained less weight than SHAM (Figure 1).

### Effect of Probiotics on Alveolar Bone Loss

**FIGURE 2:**
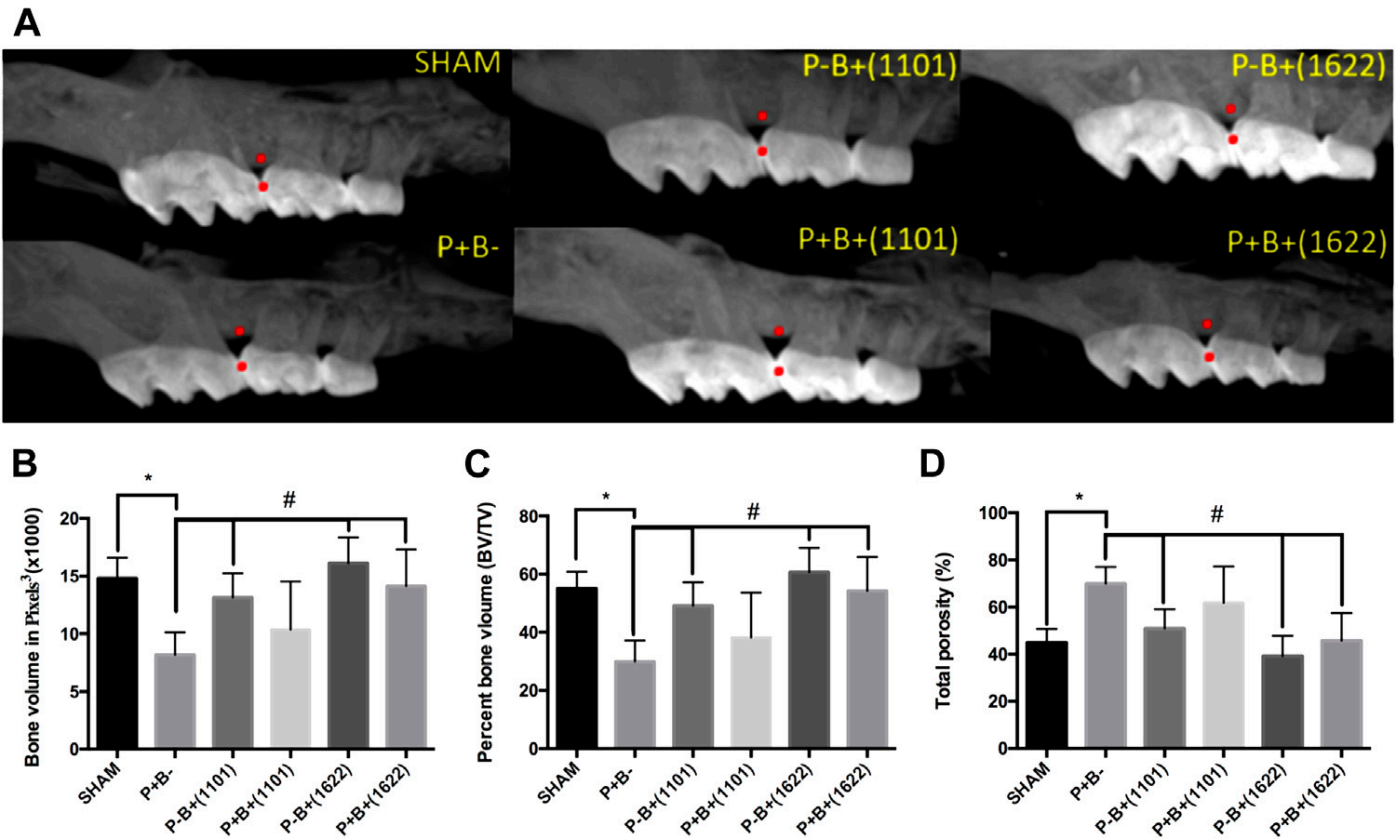
Alveolar bone analysis determined by Microtomography in the interproximal region of first and second M at the right maxilla of C57Bl/6 mice submitted to different treatments for 45 days: SHAM (negative control), P + B- (positive control), P-B+ (1101) (*B. breve* 110^1A^), P + B+ (1101) (microbial consortium + *B. breve* 110^1A^), P-B+ (1622) (*B. bifidum* 162^2A^) and P + B+ (1622) (microbial consortium + *B. bifidum* 162^2A^). **(A)** Representative images of alveolar bone. All data were obtained in the region between the red points. Data on Alveolar bone volume (ABV) (Average and sd) determined in pixels^3^ **(B)**, Percentage of alveolar bone volume (Average and sd) and **(C)** Percentage of total porosity (Average and sd) **(D)** of the different groups. * Statistically significant difference in relation to negative control (SHAM), # Statistically significant difference in relation to positive control (P + B-). ANOVA, Tukey’s multiple comparison, *p* <0.05%. Data representative of two independent experiments (*n* = 8 mice/per group).

Alveolar bone volume was determined at the interproximal region of first and second M at the left maxilla (Figure 2A). Data on bone volume, percentage of bone volume, and bone porosity in the studied groups are shown in Figures 2B–D, respectively. The microbial consortium was able to induce significant bone loss, indicated by reduced bone volume and increased porosity in positive control (P + B-) compared to SHAM (P-B-). Alveolar bone volume, percentage of alveolar bone volume, and bone porosity of the groups receiving only bifidobacteria [P-B+ (1101) and P-B+ (1622)] did not differ from SHAM (ANOVA, *p* > 0.05), indicating that *B. bifidum* 162^2A^ and *B. breve* 110^1A^ did not induce alveolar bone loss. Administration of *B. bifidum* 162^2A^ prevented the reduction in bone volume and increase in bone porosity induced by the microbial consortium [P + B+ 1622) ≠ P + B-, *p* < 0.05], whereas administration of *B. breve* 110^1A^ did not.

### Gene Expression in the Gingival Tissue

**FIGURE 3:**
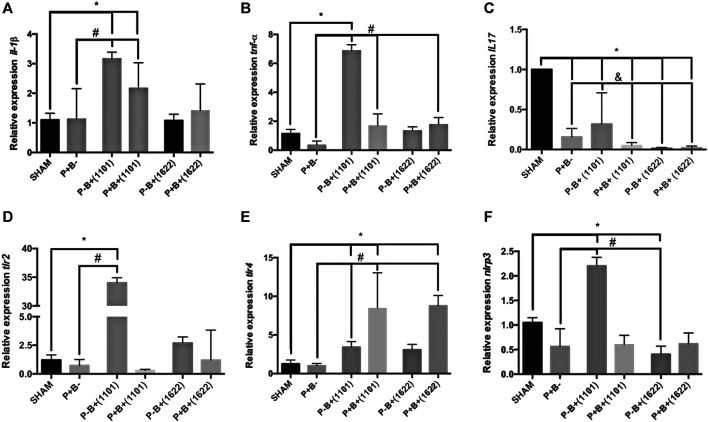
Bifidobacteria alter transcription of genes encoding cytokines and receptors for PAMPS in gingival tissues. Relative transcription of *Il-1β* **(A)** and *Tnf-α* **(B)**, *Il-17* **(C)**, *Tlr2* **(D)**, *Tlr4* **(E)** and *Nrlp3* **(F)**, determined by RT-qPCR in gingival tissues of C57Bl/6 mice submitted to different treatments for 45 days of experimental period: SHAM (negative control), P + B- (positive control), P-B + (1101) (*B. breve* 110^1A^), P + B+ (1101) (microbial consortium + *B. breve* 110^1A^), P-B+ (1622) (*B. bifidum* 162^2A^) and P + B+ (1622) (microbial consortium + *B. bifidum* 162^2A^). * Statistically significant difference in relation to negative control (SHAM), # Statistically significant difference in relation to positive control (P + B-). & Statistically significant difference in relation to P-B+ (1101). ANOVA, Tukey’s multiple comparison, *p* <0.05%. Data representative of two independent experiments (*n* = 8 mice/per group).

Despite the microbial consortium induced bone destruction, there were no differences in mRNA levels of *Il-1β* and *Tnf-α* or of genes encoding receptors for PAMPs between P + B- and SHAM groups, as shown in Figures 3A,B. However, transcription of *Il-17* was down-regulated in P + B- compared to SHAM (Figure 3C). The daily administration of *B. breve* 110^1A^ up-regulated transcription of *Il-1β* and *Tnf-α* [P-B+ (1101) and P + B+ (1101) ≠ SHAM], Figures 3A,B. All treatments downregulated transcription of *Il-17* when compared to SHAM, but the group treated only with *B. breve* 110^1A^ [P-B+(1101)] showed the highest *Il-17* mRNA levels among the treated groups (Figure 3C). The microbial consortium down-regulated transcription of *Il-1β*, *Tnf-α* and *Il-17* in animals receiving *B. breve* 110^1A^ [P + B+(1101) ≠ P-B+(1101)], although *Il-1β* and *Tnf-α* transcript levels in P + B+(1101) were still above those of the P + B- group*.* Meanwhile, the oral administration of *B. bifidum* 162^2A^ did not interfere in *Il-1β* regulation, down-regulated *Il-17*, but promoted a slight up-regulation (less than 2 folds) in *Tnf-α* transcription [P + B+ 1622) ≠ P + B-], Figure 3.

The microbial consortium did not alter the transcription profile of genes encoding receptors to PAMPs in the gingival tissues (Figures 3D–F). However, transcription of *Tlr2*, *Tlr4*, and *Nrlp3* was up-regulated by *B. breve*110^1A^ [P-B+ (1101) ≠ P + B- ≠ SHAM]. The positive regulation of *Tlr2* and *Nrlp3* was attenuated by the microbial consortium [P + B+ (1101)]. On the other hand, both *Bifidobacterium* strains induced up-regulation of *Tlr4* in mice that were also challenged with the microbial consortium [P + B+ (1101) and P + B+ 1622) ≠ P + B- ≠ SHAM], Figure 3E.

### *P. gingivalis* Levels in Oral Biofilm

*P. gingivalis* levels were determined by amplification of *16SrRNA* using species-specific primers, and data were normalized by CT values in SHAM. Oral inoculation of microbial consortium did not induce *P. gingivalis* persistent colonization of the oral biofilm*,* since no group reached CT values above background.

### Tregs and Th17 Populations

**FIGURE 4:**
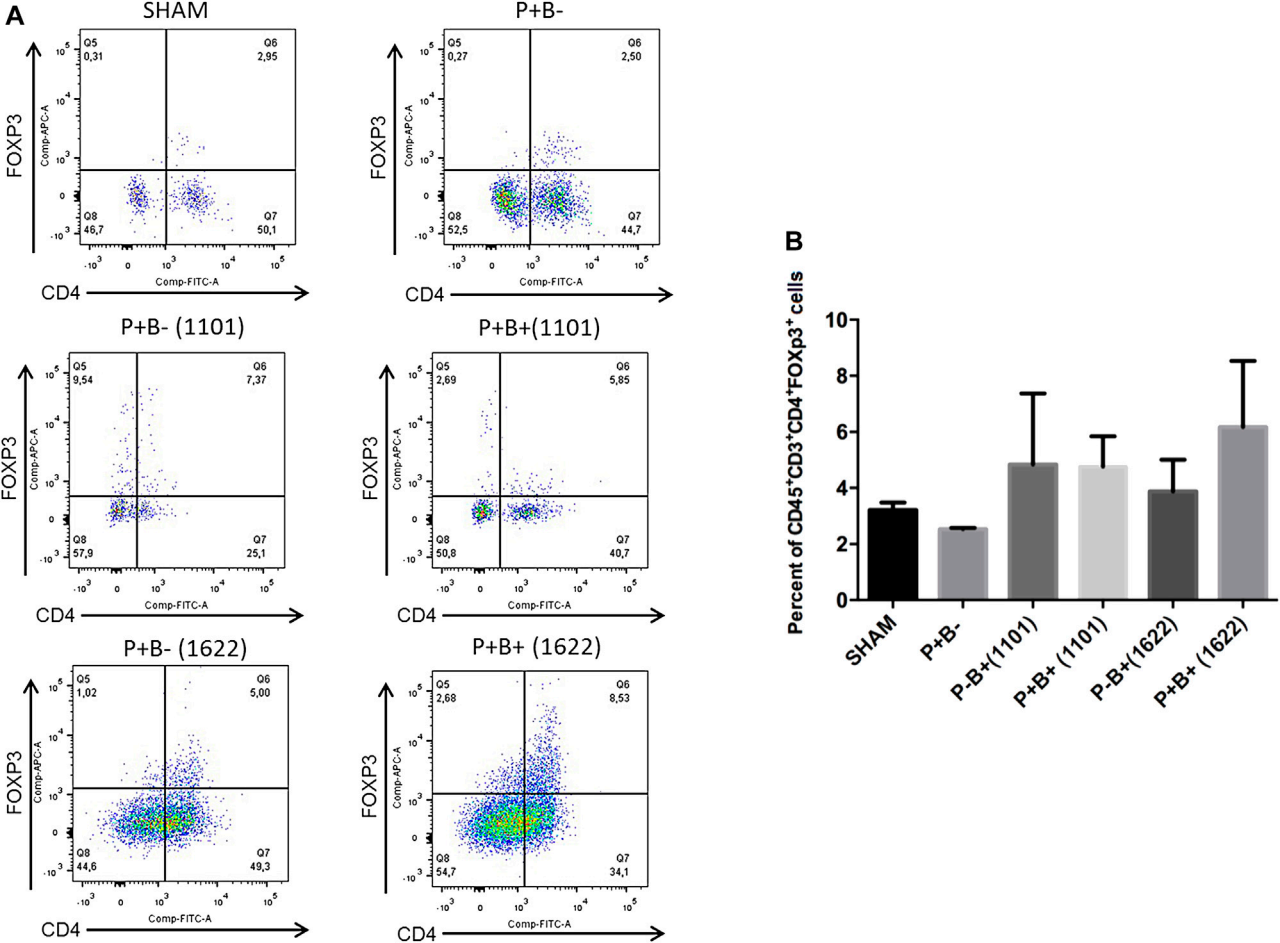
Treg populations remained unchanged in gingival tissues of C57Bl/6 mice submitted to different treatments: SHAM (negative control), P + B- (microbial consortium), P-B+ (1101) (*B. breve* 110^1A^), P + B+ (1101) (microbial consortium + *B. breve* 110^1A^), P-B+ (1622) (*B. bifidum* 162^2A^) and P + B+ (1622) (microbial consortium + *B. bifidum* 162^2A^). In **(A)** representative flow cytometry diagram showing the gating of CD4^+^, FoxP3+ Treg cells. In **(B)** Average percentages of CD4^+^, FoxP3+ Treg cells. No differences among the groups. ANOVA, Tukey’s multiple comparison, *p*>0.05%. Facs plots represent the results of one of two independent experiments with similar results (*n* = 2 pooled samples from 4 mice/per group).

**FIGURE 5:**
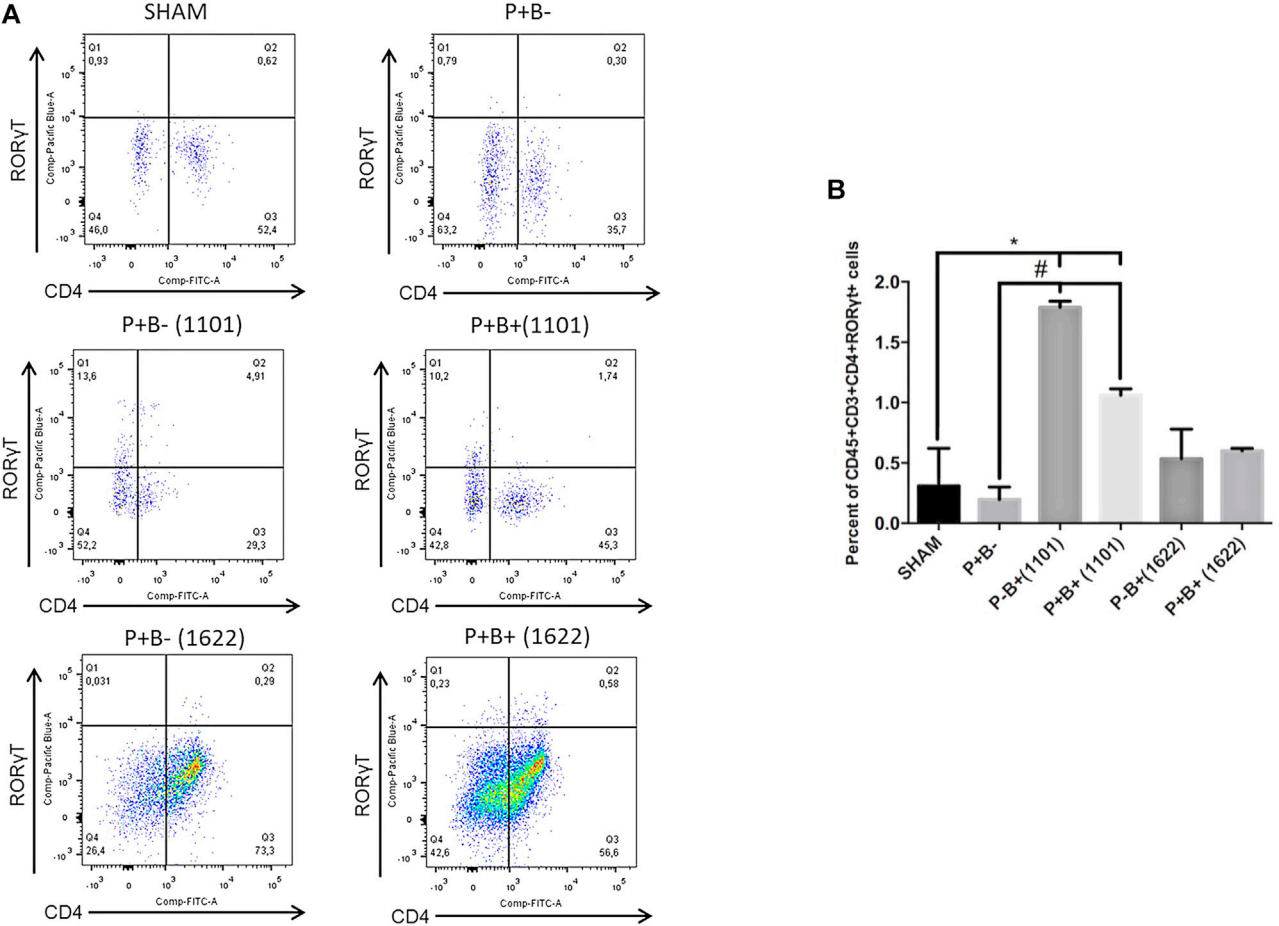
Oral administration of *B. breve* 110^1A^ increases Th17 population in the gingival tissue. Th17 population in the gingival tissue of C57Bl/6 mice submitted to different treatments: SHAM (negative control), P + B- (microbial consortium), P-B+ (1101) (*B. breve* 110^1A^), P + B+ (1101) (microbial consortium + *B. breve* 110^1A^), P-B+ (1622) (*B. bifidum* 162^2A^) and P + B+ (1622) (microbial consortium + *B. bifidum* 162^2A^). In **(A)** representative flow cytometry diagram showing the gating of CD4^+^, RORγt^+^ Th17 cells. In **(B)** Average percentages of CD4^+^, RORγt^+^ Th17 cells. *Statistically significant difference in relation to negative control (SHAM), # Statistically significant difference in relation to positive control (P + B-). ANOVA, Tukey’s multiple comparison, *p*<0.05%. Facs plots represent the results of one of two independent experiments with similar results (*n* = 2 pooled samples from 4 mice/per group).

Immune cells were evaluated by flow cytometry analysis. Gingival tissue samples from four mice were pooled, making two pooled samples/group, due to low amount of tissue. T-helper (Th) cells (CD4^+^) were stained for CD45^+^CD3^+^CD4^+^FoxP3^+^ (Tregs cells) and for CD45^+^CD3^+^CD4^+^RORγt^+^ (Th17 cells). The percentages of innate immune cells detected are shown in the Supplementary Table S1. Percentages of Tregs or Th17 populations were similar in gingival tissues of groups P + B- and negative control (SHAM). Treg cells populations were also similar in all experimental groups (Figure 4). The administration of *B. bifidum* 162^2A^ did not induce changes in Th17 cells population. However, the oral administration of *B. breve* 110^1A^ increased CD45^+^CD3^+^CD4^+^RORγt^+^ Th17 cells population from 0.31% in SHAM gingival tissue samples to 1.79% in P + B+ (1101) and 3% in P-B+ (1101), indicating that oral inoculation of *B. breve* 110^1A^ induces a Th17 response (Figure 5).

### Serum Cytokines Levels

**FIGURE 6:**
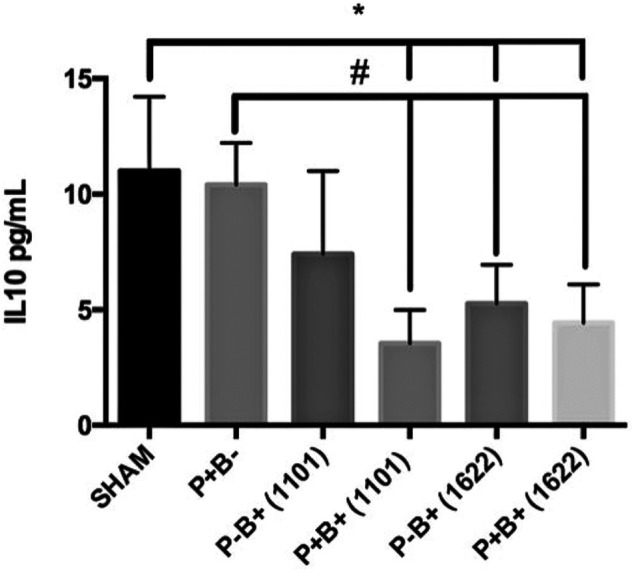
Serum levels (pg/ml) of IL-10 in C57Bl/6 mice submitted to different treatments for 45 days: SHAM (negative control), P + B- (positive control), P-B+ (1101) (*B. breve* 110^1A^), P + B+ (1101) (microbial consortium + *B. breve* 110^1A^), P-B+ (1622) (*B. bifidum* 162^2A^) and P + B+ (1622) (Microbial consortium + *B. bifidum* 162^2A^). * Statistically significant difference in relation to negative control (SHAM), # Statistically significant difference in relation to positive control (P + B-). ANOVA, Tukey’s multiple comparison, *p* <0.05%. Data representative of two independent experiments (*n* = 8 mice/per group).

Serum levels of IL-10 were similar in groups P + B- and negative control (SHAM). However, the bifidobacteria oral regimen decreased serum levels of IL-10, especially in the groups that were also inoculated with the microbial consortium (Figure 6). There were no differences in TNF-α levels among groups (data not shown).

## Discussion

In the present study, the infection model with oral inoculation of *P. gingivalis, F. nucleatum*, *P. intermedia* and *S. gordonii* was efficient in reducing alveolar bone volume and increasing total bone porosity in C57Bl/6 mice. Thus, the protocol was able to induce experimental periodontitis*,* as shown in other studies (Blasco-Baque et al., 2012; Barbosa et al., 2015; Kuboniwa et al., 2017). Two strains of *P. gingivalis* were used, since multiple strains of the pathogen showing different virulence strategies occur in humans (Amano et al., 2000). Despite bone resorption, *P. gingivalis* was not detected at the end of the experimental period, and expression of *Il-1β*, *Tnf-α*, and genes encoding receptors to PAMPS, as well as the percentage of Treg and Th17 cells populations were not altered in gingival samples of the periodontitis group (P + B-) when compared to SHAM. Some studies demonstrated that the inflammatory cytokines were detected only in the initial phase of induction of periodontitis in mouse model (Polak et al., 2009; Ebbers, et al., 2018), thus for cytokine detection, early time points analysis would be required. In contrast, expression of *Il-17* was downregulated by the microbial consortium, confirming the breakage of homeostasis. These findings are in accordance with others who reported that persistent colonization of *P. gingivalis* may not be achieved, but bone loss is seen due to dysbiosis promoted by *P. gingivalis*, a keystone pathogen (Payne et al., 2019). Similarly, *P. gingivalis* strain W83 does not up-regulate transcription of *Tnf-α* and *Il-1β* in mice gingival tissues (Sato et al., 2018). Moreover, the effects of administration of bifidobacteria differed in mice challenged with the microbial consortium from control mice.

We have shown that the oral administration of *B. bifidum* 162^2A^, but not of *B. breve* 110^1A^, was able to control alveolar bone loss induced by the microbial consortium. These *in vivo* data contrast to *in vitro* results showing that *B. breve* 110^1A^ and *B. bifidum* 162^2A^ can aggregate in multispecies biofilms formed by *P. gingivalis, S. oralis* and *S. gordonii*, and reduce the abundance of *P. gingivalis* without affecting the abundance of early colonizers (Ishikawa et al., 2020). The beneficial effect of *B. breve* was also suggested by a strong antioxidant capacity of *B. breve* strain A28, which protects host cells against reactive oxygen species produced during the inflammatory process (Mendi and Aslım, 2014). However, this feature is not homogenous within this specie, and the antioxidant capacity of the studied bifidobacteria was not determined.

The benefit of bifidobacteria species in the control of periodontitis has been previously shown for *B. animalis subsp*. *lactis* HN019. The topical use of this strain prevented alveolar bone loss in rats submitted to ligature induced periodontitis (Oliveira et al., 2017). Another animal study reported the beneficial effect of *B. animalis subsp*. *lactis* HN019 even as adjunct to scaling and root planning (SRP), with concomitant reduction in the number of osteoclasts, decrease in *IL-1β* transcripts and increased expression of *IL-10* in the periodontal tissues (Ricoldi et al., 2017). Similarly, the adjunctive use of *Bifidobacterium animalis subsp*. *lactis* HN019 in humans to treat periodontitis improved periodontal clinical parameters such as probing pocket depth and clinical attachment gain, and reduced the colonization levels of *P. gingivalis*, *Treponema denticola* and other pathobionts (Invernici et al., 2018).

Herein, a distinct response to different *Bifidobacterium* species was not only seen in terms of alveolar bone loss. The groups receiving each bifidobacteria strain (P-B+), or each bifidobacteria plus the microbial consortium (P + B+) differed also in other parameters such as weight gain, expression of cytokines and receptors in the gingival tissue, and Th17 cells percentage.

*B. breve* 110^1A^ lack of effect in preventing alveolar bone loss induced by the periodontal pathogenic consortium was followed by lower weight gain and higher inflammatory response in gingival tissues when compared to the other groups. The inflammatory profile induced by *B. breve* 110^1A^ was evidenced by the upregulation of transcription of *Tnf-α*, *Il-1β*, *Tlr2*, *Tlr4*, and *Nrlp3*, a slight down-regulation of *Il-17* and increase of Th17 cells population in the gingival tissues. On the other hand, administration of *B. bifidum* 162^2A^ alone or in combination with the microbial consortium did not induce any changes, except for a substantial down regulation of *Il-17* mRNA levels and an upregulation of *TLr4* in mice challenged with the microbial consortium.

The oral administration of the two bifidobacteria also yielded different outcomes on the transcription of *Nrlp3* and *Il-1β*, suggesting their influence on inflammasome modulation. *B. breve* 110^1A^ up-regulated transcription of *Nrlp3* and *Il-1β* compared to the SHAM group, whereas *B. bifidum* 162^2A^ down-regulated *Nrlp3* mRNA levels and did not affect transcription of *Il-1β*. However, concomitant administration of the microbial consortium with the bifidobacteria resulted in decrease of the high mRNA levels of *Nrlp3* and *Il-1β* induced by *B. breve* 110^1A^ and an increase in these transcripts in the *B. bifidum* 162^2A^ group, although it did not reach the high levels achieved by *B. breve* 110^1A^. Previous *in vitro* data may help explaining the different outcomes on the control of periodontitis by the two tested bifidobacteria. *P. gingivalis* W83 challenged gingival epithelial cells showed increased viability when co-infected with *B. bifidum* 162^2A^ but not with *B. breve* 110^1A^ (Albuquerque-Souza et al., 2019). On the other hand, co-culture of *P. gingivalis* ATCC 33277 challenged GECs with *B. breve* 110^1A^ resulted in high production of IL-1β and CXCL-8 differing from infected GECs co-cultured with *B. bifidum* 162^2A^ (Albuquerque-Souza et al., 2019).

It is well known that inflammasome activation differs according to the challenging bacteria species, target cells and environmental conditions including periodontal tissues (Taxman et al., 2012; Okano et al., 2018; Aral et al., 2021). Thus, the variable regulation of Nrlp3 and Il-1β induced by the two bifidobacteria may play a role on their effects in periodontal tissues. Tissue destruction in periodontitis is associated with the positive regulation of inflammasome-associated receptors such as NRLP3 (Xue et al., 2015) and production of IL-1β (Silva et al., 2015). Thus, induction of *Il-1β* and *Nrlp3* transcription in gingival tissues by *B. breve* 110^1A^ under a commensal microbiome is indicative of its pro-inflammatory activity. In contrast, *B. bifidum* 162^2A^ may partially surpass pathogen´s strategy to inhibit inflammasome activation in order to evade host defenses (Taxman et al., 2012; Okano et al., 2018) since its administration in the group receiving the microbial consortium attenuated *Nrlp3* down-regulation.

Administration of *B. breve* 110^1A^ without any other challenge up-regulated the transcription of *Tlr*4 and *Tlr2*. Up-regulation of *Tlr2* was previously shown for other probiotics (Li et al., 2019). *Tlr4* mRNA levels increased with concomitant administration of the microbial consortium and *B. breve* 110^1A^ or B. *bifidum* 162^2A^. Bifidobacteria are known to upregulate *Tlr4* due to production of lactic acid (Kanmani et al., 2019). *B. breve* 110^1A^ induced upregulation of PRRs under a symbiotic microbiome suggests increased recognition of commensals, and pro-inflammatory down-stream cascades, as indicated by increased expression of *Tnf-α* and *Il-1β* in the P-B+1101 group. However, *Tlr4* up-regulation promoted by both *Bifidobacterium* under pathogens challenge may increase pathogen’s recognition, and elimination. In contrast, down-regulation of *Tlr2* under *P. gingivalis* challenge may be beneficial, since TLR2 signaling in immune cells impairs their phagocytic activity, which promotes pathogen`s survival (Maekawa et al., 2014).

Regulatory T lymphocytes (Tregs) play a fundamental role in the control of inflammatory response, suppressing the proliferation and cytokine production of effector T cells, especially Th1 and Th17 (Gonzales, 2015). However, Treg populations in the gingival tissues of mice were not altered by the different treatments, suggesting this mechanism is not induced by the studied bifidobacteria. Oral inoculation of *B. breve* 110^1A^ altered T cells population in gingival tissues, leading to increased percentages of Th17, which was partially attenuated by the microbial consortium. Transcription of *Il-17* was demonstrated in the gingival tissue of the non-infected control mice, but all treatments with the microbial pathogenic consortium and/or the bifidobacteria down-regulated *Il-17* transcript levels. However, *Il-17* mRNA levels were higher in the group receiving *B. breve* 110^1A^, whereas *B. bifidum* 162^2A^ induced the lowest levels among all groups. The inflammatory functions of Th17 cells depend on the different combinations of cytokines expressed in the environment (Bunte and Beikler, 2019) and these cells present multiple functions. While IL-17 production is key to homeostasis, Th17 exacerbated activation by microbial challenge can be deleterious (Moutsopoulos et al., 2012). Thus, Th17 cell analyses also indicated that *B. breve* 110^1A^ induces an inflammatory profile in gingival tissues, differing from *B. bifidum* 162^2A^.

These data should be taken under the limitations of the mice model where *P. gingivalis* persistent colonization was not achieved. Furthermore, the data were obtained at a single time point, 12-days after the last inoculation of the microbial consortium, whereas bifidobacteria were administered throughout the 45-days experimental period. It is also worthy to emphasize that the immune cells evaluation was performed with only two pooled samples per group, to minimize the number of mice, but the data were reproducible in two independent assays.

Overall, our data showed that the beneficial effect of *Bifidobacterium* to the periodontal tissues is not a common trait of this genus. The pro-inflammatory effect of *B. breve* 110^1A^ in gingival tissues may indicate increased defenses against invading organisms but also brings some concern on the safety of this species. Our data are corroborated by others reporting bacteremia by bifidobacteria, including *B. breve* (Esaiassen et al., 2017), even when used as a probiotic (Sato et al., 2016). Therefore, considering that probiotics outcomes depend on factors related to the host and their microbiome, their outspread use should be rethought. On the other hand, *B. bifidum* 162^2A^ is a potential candidate as a probiotic to control periodontitis. *B. bifidum* 162^2A^ did not lead to significant changes in inflammatory parameters and prevented alveolar bone loss without noticeable side effects. Further studies are still needed before its clinical indication.

## Data Availability

The original contributions presented in the study are included in the article/Supplementary Material, further inquiries can be directed to the corresponding author.
