# Supplementary material for: Bifidobacterium Strains Present Distinct Effects on the Control of Alveolar Bone Loss in a Periodontitis Experimental Model
Source: Front Pharmacol. 2021 Sep 24;12:713595. doi: 10.3389/fphar.2021.713595 (PMC8497694; doi:10.3389/fphar.2021.713595)
Supplement: Supplementary file 1 [file Table1.pdf]

Table 1. Live innate immune cells (mean percentage ± sd) according to expression of receptors in the gingival tissue of C57Bl/6 mice submitted to different treatments for 45 days. SHAM (negative control), P+B- (positive control), P-B+ (1101) (*B. breve* 110^{1A}), P+B+ (1101) (microbial consortium + *B. breve* 110^{1A}), P-B+ (1622) (*B. bifidum* 162^{2A}) and P+B+ (1622) (microbial consortium + *B. bifidum* 162^{2A}).

**GINGIVAL TISSUE IMMUNE CELLS**	**SHAM**	**P+B-**	**P-B+(1101)**	**P+B+(1101)**	**P-B+(1622)**	**P+B+(1622)**
LIMPHOCYTES	10.8 ± 3.4	25.8 ± 10.3	39.6 ± 6.4$^{*\#}$	37.6 ± 9.3$^{*\#}$	35.5 ± 5*	54.4 ± 1$^{*\#\wedge}$
$CD45^+$	25.4 ± 12.7	36.5 ± 5.8	64.5 ± 6.2$^{*\#}$	68.6 ± 13.5$^{*\#}$	33.7 ± 11.5	54.3 ± 8.0$^{*\#\wedge}$
$CD45^+CD3^+$	8.5 ± 0.1	8.49 ± 2.01	9.0 ± 1.2	10.9 ± 0.4	11.6 ± 1.3	16.8 ± 0
$CD45^+CD3^+CD4^+$	47.5 ± 6.6	43.7 ± 5.6	32.3 ± 0.9	41.8 ± 4.5$^{*\#}$	68.1 ± 5.4	52.7± 1.5
$CD45^+CD3^+CD4^-$ $ROR\gamma t^+$	0.4 ± 0.4	0.4 ± 0.3	9.2 ± 4.3	10.2 ± 0.0	0.02 ± 0.0	0.1 ± 0.03
$CD45^+CD3^+CD4^+$ $ROR\gamma t^+$	0.3 ± 0.3	0.1 ± 0.1	3 ± 1.9	1.7 ± 0.05	0.5 ± 0.2	0.6 ± 0.02
$CD45^+CD3^+CD4^+$ $ROR\gamma t^-$	45.7 ± 6.6	41.4 ± 5.7	34.1 ± 4.8	41.7 ± 3.5	68.3 ± 5$^{*\#}$	55 ± 1.6
$CD45^+CD3^+CD4^-$ $ROR\gamma t^-$	53.4 ± 7.4	57.9 ± 5.2*	53.6 ± 1.4	46.2 ± 3.4$^{\&}$	31.1 ± 4.7$^{*\#}$	44.2 ± 4.1$^{*\wedge}$
$CD45^+CD3^+CD4^-$ $FOXp3^+$	0.5 ± 0.2	0.9 ± 0.7	6.2 ± 3.3	3.7 ± 1.0	0.7 ± 0.2	2.27 ± 0.41
$CD45^+CD3^+CD4^+$ $FOXp3^+$	3.2 ± 0.2	2.5 ± 0.04	4.8 ± 2.5	4.7 ± 1.0	3.8 ± 1.1	6.1 ± 2.3
$CD45^+CD3^+CD4^+$ $FOXp3^-$	42.9 ± 7.2	39.0 ± 5.6	28.5 ± 3.4	37.6 ± 3.1	56.8 ± 7.5$^{\#}$	37.9 ± 3.8$^{\wedge}$
$CD45^+CD3^+CD4^-$ $FOXp3^-$	53.3 ± 6.6	57.4 ± 4.9	60.4 ± 2.5	53.9 ± 3.1	38.5 ± 6.1$^{\#}$	53.6 ± 1.1

* Statistically significant difference in relation to negative control, # Statistically significant difference in relation to positive control P+B-, & Statistically significant difference in relation to the group P-B+ (1101), ^ Statistically significant difference in relation to the group P-B+ (1622). ANOVA, Tukey's multiple comparison, p <0.05%.
